# Supplementary material for: Evaluation of Polyphenol Intake in Pregnant Women from South-Eastern Spain and the Effect on Anthropometric Measures at Birth and Gestational Age
Source: Nutrients. 2024 Sep 13;16(18):3096. doi: 10.3390/nu16183096 (PMC11435302; doi:10.3390/nu16183096)
Supplement: Supplementary file 1 [file nutrients-16-03096-s001.zip › Supplementary File S1 Results by subfamily of phenolic compounds.docx]

| Polyphenols (mg/day) | 1^st^ Trimester | | | | | 3^rd^ Trimester | | | | | Average | | | | |
| --- | --- | --- | --- | --- | --- | --- | --- | --- | --- | --- | --- | --- | --- | --- | --- |
| **Flavonoids Group** | **Mean** | **SD** | **P_25_** | **P_50_** | **P_75_** | **Mean** | **SD** | **P_25_** | **P_50_** | **P_75_** | **Mean** | **SD** | **P_25_** | **P_50_** | **P_75_** |
| Anthocyanins | 93.6 | 66.9 | 46.9 | 76.8 | 122.3 | 75.4 | 78.9 | 28.4 | 52.9 | 93.8 | 93.6 | 66.9 | 46.9 | 76.8 | 122.3 |
| Chalcones | 0.009 | 0.009 | 0.002 | 0.008 | 0.012 | 0.007 | 0.009 | 0 | 0.003 | 0.016 | 0.009 | 0.009 | 0.002 | 0.008 | 0.012 |
| Dihydrochalcones | 3.32 | 2.95 | 1.23 | 2.42 | 4.55 | 3.21 | 3.64 | 0.92 | 1.86 | 4.87 | 3.32 | 2.95 | 1.23 | 2.42 | 4.55 |
| Dihydroflavonols_Dic | 355 (52.2%) | | | | | 95 (14%) | | | | | 383 (56.3%) | | | | |
| Flavanols | 366.4 | 247.2 | 166.4 | 320.5 | 511.3 | 336.5 | 295.5 | 123.3 | 236.9 | 452.9 | 366.4 | 247.2 | 166.4 | 320.5 | 511.3 |
| Flavanones | 95.9 | 74.9 | 36.9 | 81 | 132.9 | 93.5 | 97.3 | 23.6 | 61.7 | 136.5 | 95.9 | 74.9 | 36.9 | 81 | 132.9 |
| Flavones | 15.3 | 10.8 | 8 | 12.7 | 19.5 | 14.4 | 13.5 | 5.5 | 10.5 | 19.6 | 15.3 | 10.8 | 8 | 12.7 | 19.5 |
| Flavonols | 64.5 | 34.9 | 41.9 | 58.7 | 78.3 | 58.3 | 44.3 | 32.5 | 49.8 | 72.2 | 64.5 | 34.9 | 41.9 | 58.7 | 78.3 |
| Isoflavonoids | 0.185 | 0.146 | 0.046 | 0.181 | 0.306 | 0.183 | 0.173 | 0.024 | 0.111 | 0.299 | 0.185 | 0.146 | 0.046 | 0.181 | 0.306 |
| **Lignans Group** | **Mean** | **SD** | **P_25_** | **P_50_** | **P_75_** | **Mean** | **SD** | **P_25_** | **P_50_** | **P_75_** | **Mean** | **SD** | **P_25_** | **P_50_** | **P_75_** |
| Lignans | 63.1 | 37.8 | 36.8 | 54.9 | 83.3 | 57.8 | 45.7 | 28.1 | 46.2 | 74.9 | 63.1 | 37.8 | 36.8 | 54.9 | 83.3 |
| **Phenolicacids Group** | **Mean** | **SD** | **P_25_** | **P_50_** | **P_75_** | **Mean** | **SD** | **P_25_** | **P_50_** | **P_75_** | **Mean** | **SD** | **P_25_** | **P_50_** | **P_75_** |
| Hydroxybenzoic acids | 77.1 | 51.6 | 42 | 65.2 | 96.2 | 66.3 | 66.3 | 26.9 | 48.2 | 82.9 | 77.1 | 51.6 | 42 | 65.2 | 96.2 |
| Hydroxycinnamic acids | 329.3 | 155.6 | 216.8 | 315.2 | 415.4 | 284.9 | 170.4 | 158.5 | 251.6 | 368.7 | 329.3 | 155.6 | 216.8 | 315.2 | 415.4 |
| Hydroxyphenylacetic acids | 7.33 | 8.33 | 1.18 | 5.43 | 8.26 | 6.04 | 9.03 | 1.16 | 5.40 | 10.81 | 7.33 | 8.33 | 1.18 | 5.43 | 8.26 |
| Hydroxyphenylpropanoic acids | 0.924 | 1.056 | 0.147 | 0.685 | 1.027 | 0.764 | 1.144 | 0.147 | 0.685 | 1.369 | 0.924 | 1.056 | 0.147 | 0.685 | 1.027 |
| **Stilbenes Group** | **Mean** | **SD** | **P_25_** | **P_50_** | **P_75_** | **Mean** | **SD** | **P_25_** | **P_50_** | **P_75_** | **Mean** | **SD** | **P_25_** | **P_50_** | **P_75_** |
| Stilbenes | 0.336 | 0.309 | 0.138 | 0.255 | 0.429 | 0.201 | 0.246 | 0.061 | 0.127 | 0.244 | 0.336 | 0.309 | 0.138 | 0.255 | 0.429 |
| **Other Polyphenols Group** | **Mean** | **SD** | **P_25_** | **P_50_** | **P_75_** | **Mean** | **SD** | **P_25_** | **P_50_** | **P_75_** | **Mean** | **SD** | **P_25_** | **P_50_** | **P_75_** |
| Alkylmethoxyphenols | 0.5 | 0.467 | 0.082 | 0.421 | 0.769 | 0.419 | 0.538 | 0.005 | 0.212 | 0.685 | 0.5 | 0.467 | 0.082 | 0.421 | 0.769 |
| Alkylphenols | 12.9 | 16 | 1.3 | 4.9 | 20.4 | 13.6 | 20.6 | 0.8 | 2.1 | 26.1 | 12.9 | 16 | 1.3 | 4.9 | 20.4 |
| Furanocoumarins | 0.028 | 0.024 | 0.011 | 0.02 | 0.04 | 0.025 | 0.035 | 0.01 | 0.02 | 0.02 | 0.028 | 0.024 | 0.011 | 0.02 | 0.04 |
| Hydroxybenzaldehydes | 0.045 | 0.059 | 0.015 | 0.026 | 0.054 | 0.02 | 0.024 | 0.006 | 0.014 | 0.024 | 0.045 | 0.059 | 0.015 | 0.026 | 0.054 |
| Hydroxybenzoketones_Dic | 368 (54.1%) | | | | | 315 (46.3%) | | | | | 418 (61.5%) | | | | |
| Hydroxycinnamaldehydes_Dic | 23 (3.4%) | | | | | 8 (1.2%) | | | | | 31 (4.6%) | | | | |
| Hydroxycoumarins | 0.024 | 0.038 | 0.002 | 0.007 | 0.038 | 0.006 | 0.018 | 0 | 0.001 | 0.005 | 0.024 | 0.038 | 0.002 | 0.007 | 0.038 |
| Methoxyphenols | 0.070 | 0.066 | 0.009 | 0.059 | 0.108 | 0.059 | 0.076 | 0 | 0.03 | 0.096 | 0.07 | 0.066 | 0.009 | 0.059 | 0.108 |
| Naphtoquinones_Dic | 67 (9.9%) | | | | | 16 (2.4%) | | | | | 80 (11.8%) | | | | |
| Tyrosols | 40.2 | 31.9 | 18.5 | 31.4 | 50.7 | 34.7 | 34.9 | 11.9 | 26 | 45.8 | 40.2 | 31.9 | 18.5 | 31.4 | 50.7 |
| Other polyphenols | 1.88 | 1.16 | 1 | 1.62 | 2.46 | 1.7 | 1.37 | 0.73 | 1.23 | 2.53 | 1.88 | 1.16 | 1 | 1.62 | 2.46 |
